# Chemical Survey and Risk Assessment of 56 Pesticides in the Sado River Estuary (Iberian Peninsula, Portugal)

**DOI:** 10.3390/toxics11050457

**Published:** 2023-05-14

**Authors:** Maria João Rocha, Eduardo Rocha

**Affiliations:** 1 Laboratory of Histology and Embryology, Department of Microscopy, School of Medicine and Biomedical Sciences Abel Salazar (ICBAS), University of Porto, 4050-313 Porto, Portugal; erocha@icbas.up.pt; 2 Team of Histomorphology, Physiopathology, and Applied Toxicology, Interdisciplinary Centre of Marine and Environmental Research (CIIMAR), University of Porto, 4050-313 Porto, Portugal

**Keywords:** Sado River, surface waters, 2013/39/EU, insecticides, herbicides, fungicides

## Abstract

The Sado basin (~8000 km^2^) is an area where intensive agriculture occurs. However, this region still has few data about the water levels of priority pesticides such as fungicides, herbicides, and insecticides. Therefore, water samples were collected every two months at nine sites along the Sado River Estuary and analyzed by GC-MS/MS to determine the influx of pesticides in that ecosystem. More than 87% of the pesticides were quantified, and 42% and 72% were above the maximum totals established by the European Directives 98/83/EC and 2013/39/EU, respectively. Fungicides (91%), herbicides (87%), and insecticides (85%) attained average annual amounts of ≈3.2 µg/L, ≈1.0 µg/L, and ≈12.8 µg/L, respectively. A mathematical approach was used to evaluate the hazard of the pesticide mixture at the maximum concentrations found in this area. The assessment identified invertebrates as the most at-risk trophic level and identified two chemicals (chlorpyriphos and cyfluthrin) as the primary culprits. This assumption was supported by acute in vivo assays using *Daphnia magna*. These observations, and the high concentrations of phosphates, indicate that the status of the Sado waters poses environmental and potential human health risks.

## 1. Introduction

Marine and freshwater pollution impacts humans and wildlife worldwide [1,2,3,4]. In agricultural activities, tons of fertilizers and pesticides are applied yearly, contaminating surface and ground waters [5,6]. Consequently, estuarine and coastal environments are consistently impacted by pesticides [7,8,9], which are toxic for non-target organisms, such as birds, fish, aquatic invertebrates, and plants [10,11,12,13].

**Figure 1:**
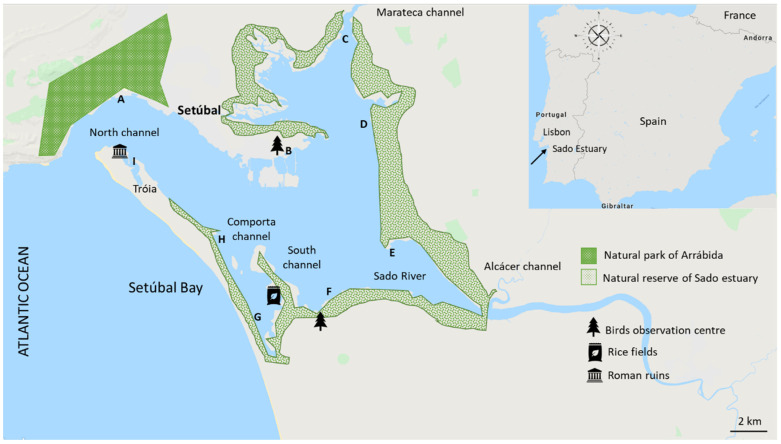
Location of the sampling sites at the Sado River estuary (southwest Iberian Peninsula, Portugal) (**A**–**I**, *n* = 9). Anthropogenic sources that may contribute to higher amounts of pesticides, such as rural areas, are referred to herein (map generated from https://mapchart.net/world.html, accessed on 10 May 2023).

The Sado River, chosen here as a case study, holds Portugal’s second-largest estuary (235 km^2^). It has ecologically critical intertidal areas, complex bathymetry, and several channels separated by sandbanks [14]. This estuary’s primary source of fresh water is the Sado River, entering through the Alcácer Channel. However, smaller streams, such as the Marateca Channel, also discharge into the estuary (Figure 1).

The Sado estuary is a legally defined natural reserve covering approximately 23,160 ha [14] with indigenous vegetation and perfect habitats for many aquatic species, such as molluscs, crustaceans, fish, and the emblematic bottlenose dolphin (*Tursiops truncates*). In addition, this area and the river areas are also widely inhabited by many species of birds, such as herons, white swans, flamingos, river birds, ducks, and birds of prey, as well as reptiles, amphibians, and other mammals, such as the European otter and bucks [14].

Unfortunately, discharges from both urban and industrial sewages and the continuous output from the Sado River (aggregating agricultural, domestic, and industrial sources from the river basin or its tributaries) contribute to the anthropogenic pressure of the respective estuary [15,16]. Rising levels of nutrients, eutrophication, pesticides and biocides, heavy metals, organotins, polychlorinated biphenyls (PCBs), polycyclic aromatic hydrocarbons (PAHs), and even natural and pharmaceutical estrogens have been reported in the Sado-associated ecosystems during recent decades [15,17,18]. These pollutants and other human activities, such as shipping and global warming, have promoted severe pressure and continuous species’ declines over the past decades (well-registered for a few, such as the snouted seahorse, bottlenose dolphin, and, recently, waterbirds) [19,20,21,22].

Additionally, past information from governing and regulatory bodies—the European Commission Database Regulation (EC No. 1107/2009) [23] and the Portuguese Regional Directorate of Agriculture and Fisheries [24]—and the lack of systematic monitoring leads us to conjecture that significant concentrations of diverse types of pesticides may exist in the Sado Estuary. This idea is reinforced by the abundance of pesticides previously found in neighboring habitats, such as the Tagus Estuary [25], which is located only approximately 24 km north of the Sado.

The present study intends to uncover the existence of up to 56 pesticides (fungicides, herbicides, and insecticides) in the Sado Estuary surface waters. These compounds were selected considering official databases [23] and represent a panel of authorized, unauthorized, and banned pesticides still in use in Portugal [24]. The new data are not only of local relevancy but also integrate efforts in the Southwest of the Iberian Peninsula to characterize the presence of pesticides and physicochemical water-quality parameters in surface waters, which levels should be following Directives 98/83/CE and 2013/39/EU [26,27]. The study also intends to investigate those pollutants’ seasonal and spatial distribution profiles and the potential environmental risks. Thus, several mathematic tools were used to represent two-dimensional figures of specific pesticides, conduct multivariate analysis using principal component analysis (PCA), and evaluate risk. The latest goal included a two-tiered computational strategy based on concentration addition (CA), independent action (IA), and in vivo toxicity assays with the water flea *Daphnia magna*. These tests intend to support the theoretical data and reveal new insights into the potential toxic effect of pesticide mixtures on the most impacted trophic level.

## 2. Materials and Methods

### 2.1. Study Area

The Sado River is located on the southwest coast of Portugal, and its basin of 7692 km^2^ holds intensive agricultural practices [24]. The Sado Estuary is part of the Arrábida Natural Park and constitutes a legally protected reserve. Nine sampling stations were considered for this study: Areas **A** to **E** at the north margin and **F** to **I** at the south (Figure 1).

Sampling station **A** (38°30′38.9″ N 8°54′55.8″ W) is located at the outer estuary, where it receives the influence of the North Channel (Figure 1) and where all pollutants from the estuary finally reach the Atlantic Ocean. Site **B** is a bird observation center (38°29′14.7″ N 8°47′01.4″ W). The sampling sites **C** (38°34′10.2″ N 8°44′08.5″ W), **D** (38°30′12.3″ N 8°43′35.8″ W), and **E** (38°26′21.5″ N 8°42′55.6″ W) are at the inner part of the estuary. Site **C** receives the influence of the Marateca Channel, which, together with the Sado River, is one of the highest contributors to the Sado Estuary. Site **F** (38°25′04.4″ N 8°42′53.2″ W) receives the impact of the Alcaçer Channel, the Sado River, and the so-called “South Channel”. Sites **G** (38°23′29.9″ N 8°48′00.8″ W) and **H** (38°25′04.1″ N 8°48′55.2″ W) are within a small peninsula, which receives the influence of the Comporta Channel. Site **I** (38°29′08.3″ N 8°53′11.5″ W) is within the Tróia Peninsula, where luxury hotel complexes exist.

### 2.2. Water Collection

The samplings occurred in the winter (February), spring (April), summer (June and September), and autumn (November and December) of 2022. Sampling was at low tide, as recommended explicitly for this estuary to evaluate worst-case scenarios in its water quality [28]. The water was collected at a 1 m depth (*n* = 6 sampling campaigns × 9 sampling sites) and inserted into pre-rinsed amber glass bottles (1.5 L each) [29]. The samples were transported to the laboratory in the dark and at 4 °C. Then, they were filtered (0.45 µm glass fiber filter), and their pH was adjusted to 7 with H_2_SO_4_ (to inhibit the degradability of the target pesticides). A solid-phase extraction (SPE) protocol (referred to below) was implemented 24 h after the field collection [29].

### 2.3. Physicochemical Parameters

Temperature (°C), dissolved oxygen (DO; mg/L), and salinity were measured in situ using a portable meter OXi 330i. The levels of nitrites (NO_2_^−^), nitrates (NO_3_^−^), ammonium (NH_4_^+^), and phosphates (PO_4_^2−^) were measured by photometry with a portable colorimeter (pHotoFlex**^®^** STD, WTW); kit 252019 (NO_2_^−^), kit 250440 (NO_3_^−^), kit 252027 (NH_4_^+^), kit 250447 (PO_4_^2−^). All samples and blanks were analyzed in triplicate to ensure precision.

### 2.4. Materials and Chemicals for GC-MS/MS Analyses

Glass fiber filters (0.45 μm) were from Munktell (Bärenstein, Germany). Ultrapure water was obtained from a Milli-Q water system (conductivity = 0.054 μS cm/L, at 25 °C). The extraction cartridges, OASIS**^®^** hydrophilic-lipophilic balance (HLB), 6 cc, were from Waters Corporation (Milford, MA, USA). Ethyl acetate, hexane, and methanol were from Romil (Cambridge, UK). All 56 pesticides were from Sigma-Aldrich (Steinheim, Germany), and apart from Mix A (EPA 505/525, 500 mg/L) and Mix B (EPA 505/525, 500 mg/L), all were bought as individual standards. The reference standards were above 98% purity, prepared in MeOH (1.0 mg/L), and kept in the dark at −20 °C to prevent decay. Both 4,4-DDT-d8 (DDT-d_8_) and atrazine-d5 (ATZ-d_5_) were used as surrogates and internal standards (IS).

### 2.5. Sample Preparation

Blanks and quality control (QC) samples were prepared using ultrapure water containing all pesticides at an intermediate concentration (160 ng/L) and IS. Water samples (500 mL) were added with 160 ng/L of DDT-d_8_ + ATZ-d_5_ and subjected to SPE for pesticide extraction and sample pre-concentration following the current methodology [30]. The OASIS HLB cartridges, adapted in an off-line SPE vacuum extraction device (Waters), were conditioned using 5 mL of ethyl acetate, 5 mL of methanol, and 2.5 mL of ultrapure water with a 1–2 mL/min flow rate. The cartridges were then filled with 500 mL of each sample at a constant flow rate (5 mL/min) and vacuum-dried (1 h). The pesticides were eluted with 6 mL of ethyl acetate at 1 mL/min, dried under a gentle N_2_ stream (99.9997%), kept in GC vials at –40 °C, and then reconstituted in 200 μL of hexane before GC-MS/MS analysis [30].

### 2.6. Quantitative Analysis

The GC-MS/MS analyses occurred in a Thermo Finnigan Electron Corporation gas chromatograph attached to an ion trap mass spectrometer (Scientific ITQ™ 1100, GC-MSn) and fitted with a Trace GOLD column (TG-5SILMS, 30 m × 0.25 mm × 0.25 µm). Injections (2 μL) of standards, blanks, QCs, and samples were performed by an autosampler in splitless mode (Thermo Scientific TriPlus™). The injector port temperature was 250 °C, and the oven temperatures were programmed as follows: (a) 65 °C (initial hold time of 2 min) to 180 °C at 20 °C/min; (b) from 180 °C to 280 °C at 5 °C/min; (c) 280 °C for 7 min. The ion source and MS transfer line were at 280 °C. The carrier gas (He, 99.9999% purity) was at a 1 mL/min constant flow rate. Details about the GC-MS/MS protocol have already been published [30] (Table S1 shows the MS/MS conditions).

### 2.7. Pesticide Hazard Assessment

Due to the difficulty of assessing the harmful actions of pesticides in aquatic environments, as these occur in different mixtures and quantities, the prediction of their environmental hazard followed a two-tiered theoretical approach, as previously implemented by our group [25].

The first tier of this theoretical tool uses the concentration addition (CA) model, which involves two consecutive steps based on the calculus of risk quotients (RQs). The first action involves the measured environmental concentrations (MEC), the assessment factor (AF), and the predictive no effect (PNEC) of each analyzed pesticide $PNEC=\frac{L\left(E\right)C50}{AF=100}$, thus $RQ=\frac{MEC}{PNEC}$ [31]. When a pesticide exhibits an RQ value > 1, it poses a risk for the studied habitat, and a second step is considered. This step involves the calculus of toxic units (TU) and then of RQs, fostered by the sum of toxic units (STU) by trophic level (algae, invertebrates, and fish):

$$TU=\frac{MEC}{L\left(E\right)C50}$$

$$RQSTU=max.\left(STUalgae,STUinvertebrates,STUfish\right)\times AF$$

If the last steps of the first tier provide RQ values > 1, then the application of the second-tier model, based on independent action (IA), is recommended [32]:

$$\frac{L\left(E\right)C50IA}{L\left(E\right)C50CA}\leq \frac{STU}{max.TUoftheselectedtrophiclevel}$$

The latest coefficient predicts when dose-additive and mixture models produce similar or divergent toxicity results and point to the number of components of a mixture responsible for that effect [32]. The values of L(E)C_50_ were obtained from FOOTPRINT and PubChem databases. However, amongst the 56 analyzed pesticides, the values of L(E)C_50_ for pentachlorobenzene (PeCB), atrazine desethyl, endosulfan sulphate, and hexachlorocyclopentadiene (HCCP) were not accessible.

### 2.8. In Vivo Toxicity Tests

The in vivo toxicity test followed the OECD guideline 202 [33], as implemented in the DaphTox F Magna ™ kit protocol (MicroBioTests, 2006). After hatching, the animals were fed spirulina before being exposed to the pesticide mixtures. Then, at least five daphnia neonate groups were placed randomly in 24 multiwell plates (www.random.org, accessed on 10 May 2023). This experiment occurred in triplicate on three different days, and the plates were maintained in the dark at 21 °C for 24 h and 48 h. The results were valid whenever the mortality of the control group was <10%.

For this bioassay, the experimental groups were as follows: **BC**—blank control; **SC**—solvent control (0.01% of EtOH); **PC**—positive control (1 mg/L K_2_Cr_2_O_7_); **Mix 1**—maximal environmental concentrations of chlorpyrifos (3.3 µg/L) and cyfluthrin (31.4 µg/L); **Mix 2**—maximal environmental concentrations of all evaluated pesticides. The number of dead organisms (i.e., no movement in 10 s of observation) established the toxicity of the mixtures.

### 2.9. Data Presentation and Statistical Analyses

When the concentrations of pesticides were below the limits of detection (LODs) of the GC-MS/MS method, these data were treated as proposed by the United States Environmental Protection Agency [34], i.e., data = $\frac{LOD}{\sqrt{2}}$). Statistical analyses used PAST 4.02 [35] and GraphPad Prism (6.01, GraphPad Software, Inc., San Diego, CA, USA) programs. To aid readers’ data interpretation, the findings in Tables are provided as the mean followed by the standard deviation (SD), whilst the graphs in Figures exhibit boxplots (with median, minimum, maximum, and 1st and 3rd quartiles). The Shapiro–Wilk W and the Levine tests were used to check the normality of datasets and the homogeneity of variances. Unidirectional analysis of variance (ANOVA) investigated differences between independent sites and groups of compounds. Tukey’s post-hoc test evaluated multiple comparisons. When the two parametric assumptions were invalid, and data transformation failed, the non-parametric Kruskal–Wallis test was used, followed by Dunn’s post hoc test. The threshold significance level for α was set at the standardized value of 0.05. At last, the PCA was performed using the correlation matrix. The principal components (PCs) were extracted considering the Kaiser (i.e., eigenvalue > 1) and the Scree Plot criteria [36,37].

## 3. Results

### 3.1. Pesticide Concentrations in the Sado Surface Waters

The average seasonal concentrations (ng/L) for each pesticide are in Table 1. This table also shows the global amounts (∑average) of each category of pesticides per season and the percentage of samples above the method detection limits (MDL), which are 91%, 87%, and 85% for fungicides, herbicides, and insecticides.

**Figure 2:**
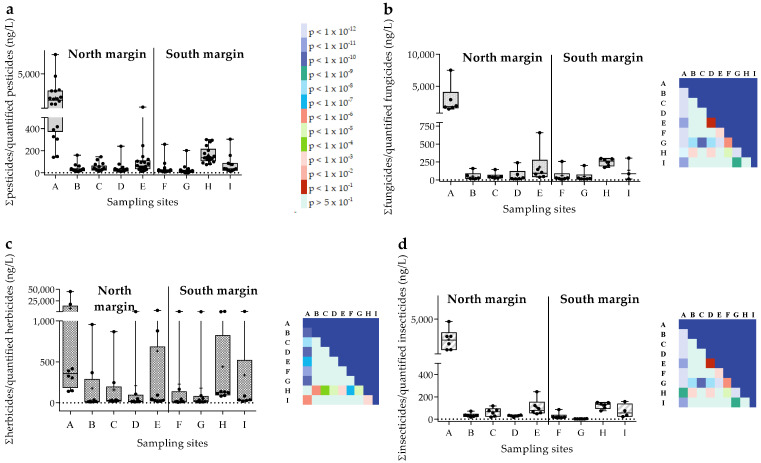
Annual levels (ng/L) of pesticides as a whole (**a**), fungicides (**b**), herbicides (**c**), and insecticides (**d**) per sampling site. Data are expressed in boxplots with the minimum, median, maximum, average (+), and interquartile range Q1–Q3. Dots represent average individual values measured at each sampling site (Fungicides *n* = 6; Herbicides *n* = 15; Insecticides *n* = 35). The color charts show statistical differences amongst sampling sites (*p* < 0.05).

Figure 2 shows the total amounts (TA) of pesticides (a), fungicides (b), herbicides (c), and insecticides (d) by sampling site at the Sado River estuary, considering its north (A–E) and south margins (F–I). This figure demonstrates that pesticide concentrations do not differ significantly amongst sampling sites (a). However, individually, each class of compounds shows higher amounts at the north margin, especially at sampling station A; all data concerning the degree of significance are illustrated in Figure 2 (the numeric details of each *p*-value are reported in supplement Table S2).

**Figure 3:**
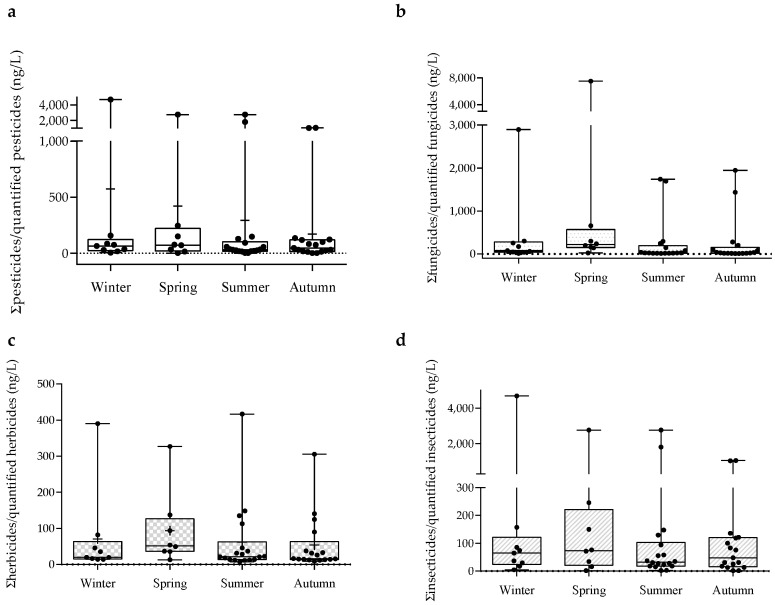
Seasonal fluctuations (ng/L) of pesticides as a whole (**a**), fungicides (**b**), herbicides (**c**), and insecticides (**d**). Data are expressed in boxplots with the minimum, median, maximum, average (+), and interquartile range Q1–Q3. Dots represent average individual values measured in each season. No statistical differences were found amongst sampling occasions.

Figure 3 reveals no significant seasonal differences amongst the concentrations of all the pesticides, fungicides, herbicides, and insecticides (*p* > 0.05).

**Figure 4:**
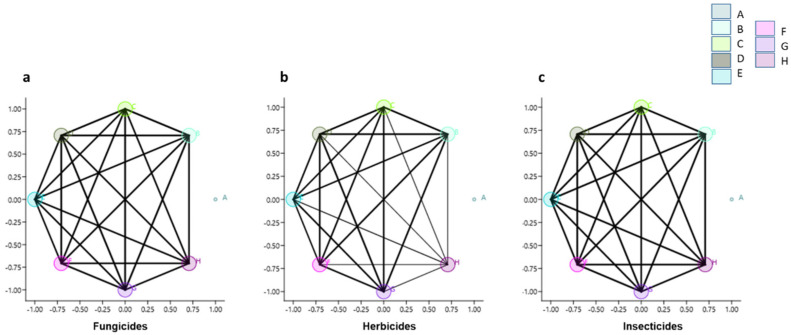
Network graph showing the interactions amongst sampling sites and seasons for (**a**) fungicides, (**b**) herbicides, and (**c**) insecticides. The larger dots and darker lines are related to a higher number and stronger interactions.

Figure 4 shows network plots for fungicides, herbicides, and insecticides. The size of nodes in the plots is proportional to the number of interactions between sampling sites and seasons. The lines link similar occurrences, and their thickness shows how strongly they are related. Sample site **A** does not show interactions with all other sampling sites and each pesticide category.

**Figure 5:**
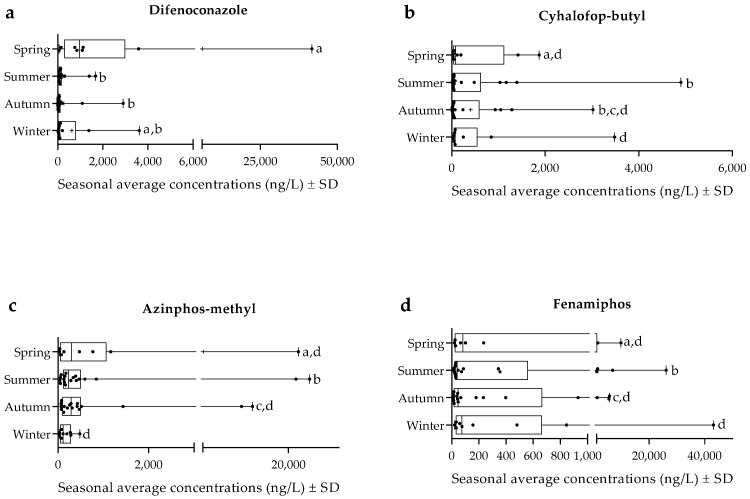
Seasonal levels (ng/L) of the pesticides with the highest concentrations within each category. (**a**) Difenoconazole, (**b**) cyhalofop-butyl, (**c**) azinphos-methyl, and (**d**) fenamiphos. Data are expressed in boxplots with the minimum, median, and maximum. Data are expressed in boxplots with the minimum, median, maximum, average (+), and interquartile range Q1–Q3. Dots represent average individual values measured in each season. Different letters refer to statistical differences (*p* < 0.05).

Figure 5 shows each category’s seasonal fluctuation patterns of the most abundant pesticides. Herein, difenoconazole was the fungicide with higher amounts, showing higher levels in spring, i.e., ≈6170 ng/L, than in the other seasons, particularly in summer and autumn (Figure 5a) (*p* < 0.05). Cyhalofop-butyl was the herbicide with higher concentrations in the Sado waters (Figure 5b). This compound showed higher amounts in summer when its average concentration was ≈470 ng/L (*p* < 0.05). The insecticides category had more investigated pesticides, and two had higher concentrations, i.e., azinphos-methyl and fenamiphos (Figure 5c,d). In spring, the average amounts measured for azinphos-methyl (c) were ≈ 3082 ng/L, whereas, in winter, those values were ≈172 ng/L (*p* < 0.05). Fenamiphos also showed seasonal trends, with higher average amounts in winter (≈4986 ng/L) (*p* < 0.05).

**Figure 6:**
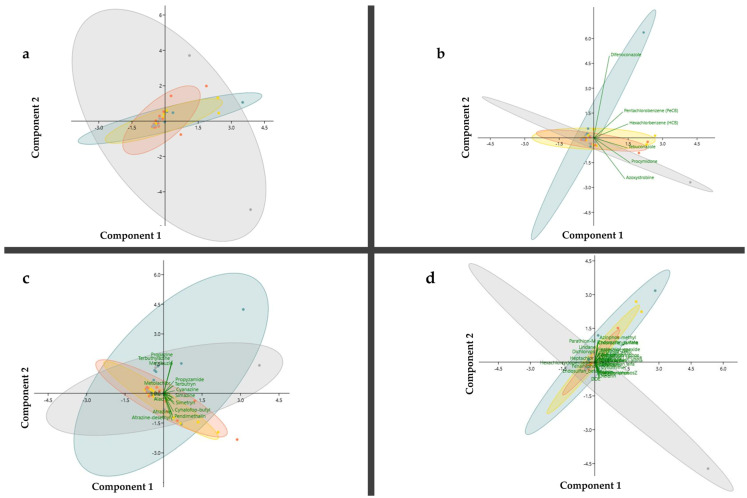
Score plots of PC1 vs. PC2 illustrating the distribution of pesticides by category season and sampling sites, i.e., all pesticides as a whole (**a**), fungicides, (**b**), herbicides (**c**), and insecticides (**d**).

PCA (Figure 6) provided qualitative comparisons amongst seasonal variabilities and the correlation of each category of pesticides among the sampling sites. This approach reveals that all assayed pesticides showed a higher dispersion of their compounds and concentrations in winter and that the most similar seasons were spring and summer (Figure 6a). PCA establishes that pesticides are characterized by eight components, which displayed 92.02% of data variance and had eigenvalues > 1.0 (data in Supplement Table S3). The main contributors of PC1 (49.2%), PC2 (20.4%), and PC3 (7.7%) were tetrachlorvinphos (0.18)—insecticide, simetryn (0.21)—herbicide, and azoxystrobin (0.23)—fungicide, respectively. The other components showed 1.79–5.9% of the total variance, and the pesticides that contributed the most to this result were metribuzin (PC4), dichlorvos (PC5), heptachlor (PC6 and PC7), and HCB (PC8).

Considering only the fungicides, this class of pesticides was characterized by two components, which displayed 80.78% of data variance and had eigenvalues > 1.0 (data in Supplement Table S2). The main contributors of PC1 were procymidone (0.48) and HCB (0.46), and that of PC2 was difenoconazole (0.82). Moreover, the 95% ellipses showed a different distribution pattern of the studied compounds demonstrating that spring and winter displayed opposite distribution patterns, whereas summer and autumn were similar (Figure 6b).

The herbicides were defined by three components that held 88.60% of the variance and showed eigenvalues > 1.0 (data in Supplement Table S2). The main contributors of PC1 (70.1%) were cyanazine (0.30) and simetryn (0.29), and those of PC2 (9.80%) were propazine (0.43) and terbuthylazine (0.43). The main contributor of PC3 (8.70%) was metolachlor (−0.48) (Table S3). The 95% ellipses show that spring and winter show different distribution patterns than summer and autumn, which were similar (Figure 6c).

Six pesticides account for 92.5% of the variance and show eigenvalues > 1.0 (data in Supplement Table S3). The main contributors of PC1 (65.25%) were deltamethrin (0.21), endosulfan-alpha (0.21), cypermethrin-alpha, and diazinon (0.20). In contrast, those of PC2 (11.93%) were azinphos-methyl (0.36), endosulfan–sulphate (0.33), and chlordane-gamma (0.30). Fenitrothion (0.37), fonofos (0.36), and phosmet (0.33) were the main contributors to PC3 (6.07%). Those of PC4 (3.52%), PC5 (2.94%), and PC6 (2.86%) were heptachlor (0.82), dichlorvos (0.82), and HCB (1.00), respectively (Table S3). The 95% ellipses show that the insecticides had similar distribution patterns in spring, summer, and autumn. In contrast, winter displayed opposite distribution patterns (Figure 6d).

### 3.2. European Regulations for Pesticides

Table 2 shows the pesticides whose average concentrations are above the maximum values set by EU Directives 98/83/EC and 2013/39/EU [26,27], as well as their values of log Kow, log Koc, and the groundwater ubiquity score (GUS). The fungicides azoxystrobin, difenoconazole, and tebuconazole concentrations were, respectively, 3.9-, 18.3-, and 4.6-fold higher than the levels established for water intended for human consumption. Moreover, the herbicides cyhalofop-butyl and metribuzin showed concentrations 4.9- and 1.3-fold higher than the limits proposed in Directive 98/83/EC. The highest average annual values were obtained for the insecticides azinphos-methyl, cyfluthrine, and fenamiphos, whose concentrations were 19.3-, 16.2-, and 23.3-fold higher than those proposed by the last decree. In summary, 50% of the fungicides, 13.3% of the herbicides, and 62.9% of the insecticides were above the limits recommended for drinking water [26]. For surface waters, 100% of the fungicides and insecticides and 20% of herbicides were above the concentrations proposed by Directive 2013/39/EU [27].

### 3.3. Evaluation of the Aquatic Hazard of Pesticide Mixtures

Table 3 shows the two-tiered approach for predicting environmental risks triggered by pesticide mixtures. In the first tier, 57% of the compounds present a MEC/PNEC ratio above 1.0, indicating their potential risk for the current environment. After that, the second tier focuses on the most sensitive group (invertebrates), suggesting that amongst all evaluated compounds, two of them (chlorpyriphos and cyfluthrin) dominate the toxicity of the present environmental mixture. In addition, the maximal STU reveals that the invertebrate trophic level is 224- and 3-fold higher than those calculated for algae and fish, respectively.

### 3.4. In Vivo Toxicity Test

**Figure 7:**
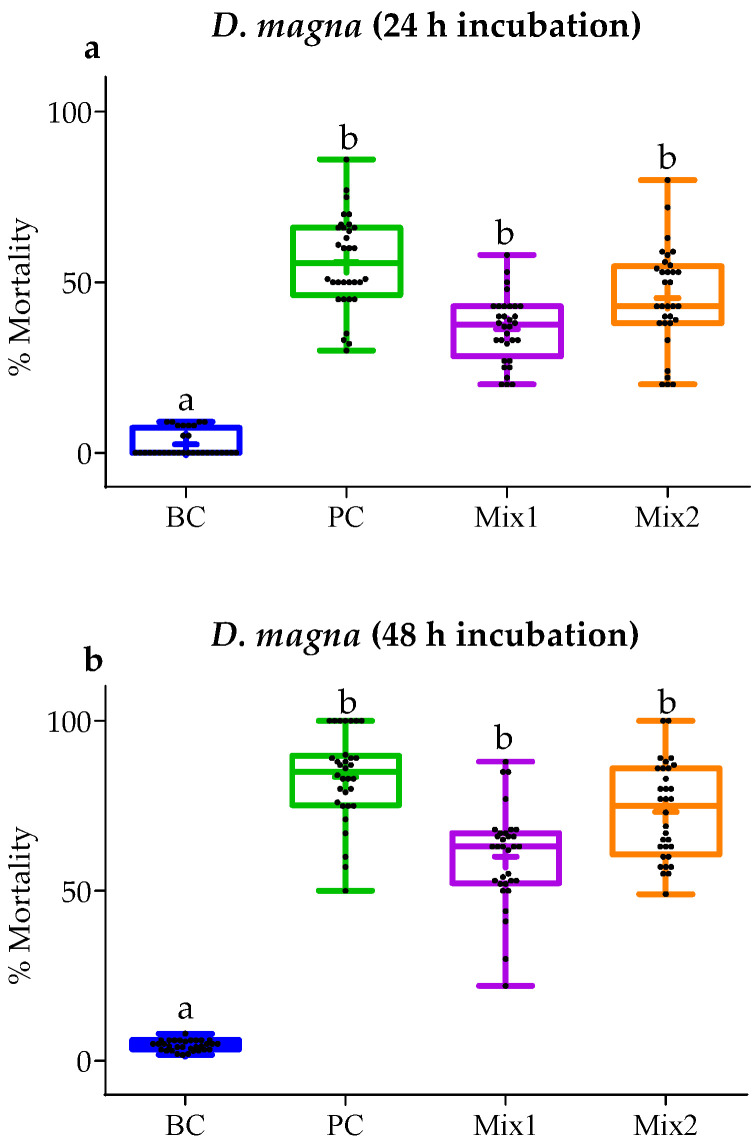
Results of *Daphnia magna* assay, given as the percentage of mortality per treatment after 24 h (**a**) and 48 h exposure (**b**). Data are expressed boxplots with minimum, median, maximum, average (+), and interquartile range Q1–Q3. Dots represent individual values (*n* = 32 for *D. magna* for each treatment): **BC**—blank control; **PC**—positive control (1 mg/L K_2_Cr_2_O_7_); **Mix 1**—MEC of chlorpyrifos and cyfluthrin; **Mix 2**—MEC of all pesticides. Different lowercase letters represent significant differences between groups (*p* < 0.05).

The assessment of standard sensitivity of *D. magna* was assessed through the calculation of K_2_Cr_2_O_7_ LC_50_ values. These were, on average, 0.9 mg/L (24 h) and 1.5 mg/L (48 h). Both values agreed with the requirements stipulated by the ISO6341 acceptability (0.6–2.1 mg/L) [38]. Figure 7 shows that for *D. magna* assays, after 24 h and 48 h of exposure, the control groups had average mortalities < 3.5% (24 h and 48 h), the pesticide **Mix 1** reached mortalities of ≈36% (24 h)–60% (48 h), and the pesticide **Mix 2** showed ≈ 46% (24 h)–73% (48 h). Significant differences between groups are shown in Figure 7 (*p* < 0.05).

### 3.5. Physicochemical Parameters

The physicochemical parameters were grouped by season and estuary margins (Table 4). The annual average levels of nitrites, nitrates, ammonia, and phosphates were 0.04 mg/L, 0.6 mg/L, 0.6 mg/L, and 0.9 mg/L, respectively. Moreover, the average amount of dissolved oxygen (DO) was 7.2 mg/L, and signs of hypoxia were never observed at any site or occasion. Table 4 also shows the annual temperature, pH, and salinity.

## 4. Discussion

The Sado River estuary is the second-largest in Portugal and has abundant agricultural practices (particularly rice crops) [39]. Such immense agrarian activity justifies regular monitoring. However, pesticide residues in water have only been subject to two overall assessments, one in 2008 [40] and one in 2017 [41].

Consequently, five years after the last survey, the present study reveals that in 2022, pesticides are still ubiquitous in the Sado River estuary. Moreover, on many occasions, their concentrations surpass those limited by the EU Directive for drinking and transitional surface waters [26,27]. This occurrence suggests an excessive application of fungicides, herbicides, and insecticides in the Sado basin, as their cumulative values in surface waters are well above the limits (0.5 µg/L) established by the Council Directive 98/83/EC [26]. Since the Sado basin (769,200 ha) is submitted to intensive agricultural production of rice, orchards, and vineyards [24], it is assumed that the pesticides’ presence in the estuary is due to their diffusion, primarily during winter floods and field irrigation [41]. This view agrees with that of EU regulatory bodies, which have defended for a long time that those contaminants’ central origin is agricultural activities [27]. It is supposed that the central origin of these contaminants continues to be agricultural activities [27]. Since the Sado basin (769,200 ha) is submitted to intensive agricultural production of rice, orchards, and vineyards [24], it is assumed that their presence here is due to their diffusion, primarily during winter floods and field irrigation [41].

Currently, considering both EU directives for individual pesticides in drinking and surface waters, herein 50–100% of fungicides, 13–20% of herbicides, and 63–100% of insecticides were in concentrations above legal recommendations [26,27]. Moreover, according to Directive 2013/39/EU [27], it is important to stress that chemicals above those thresholds are priority substances. In addition, when looking at the leaching potential of the evaluated compounds, the majority have log Kow ≥ 3. This characteristic is used as a trigger for sediment affinity and implies low leaching potentials for those compounds [42]. In this sense, fungicides such as hexachlorcyclopentadiene and pentachlorobenzene, in addition to showing concentrations 155- and 2-fold higher than those proposed by Directive 2013/39/EU [27], show a low GUS index (−1.2–0.4) and high log K_ow_ (4.0–5.2) demonstrating higher craving for organic matter than for surface water. Similar occurrences were observed for herbicides, e.g., cyhalofop-butyl (GUS index of—0.2 and a log K_ow_ of 6.0), and insecticides, e.g., cypermethrin (GUS index of -2.1 and a log Kow of 6.9). Therefore, it is assumed that these compounds’ presence in surface waters only occurs when their use is undue [43]. Such incidents, per se, should be investigated given the risks for biota, e.g., cyhalofop-butyl induces developmental toxicity and immunotoxicity in fish [44].

Surprisingly, banned insecticides, such as aldrin, DDTs, dieldrin, and mirex, are still measured in this ecosystem. Since DDT was banned in Portugal in 1990, and because of its predominance in the estuary over its degradation products (DDE, DDD), it is hypothesized that the use of dicofol and other organochlorine pesticides still in use may contain, as an impurity of manufacture, small amounts of DDT and its metabolites, which then diffuse into the estuary. The last hypothesis seems the most probable since the ratio DDT/∑DDE, DDD was ≈0.83, demonstrating the predominance of metabolites over the parent compound [45]. Different conclusions were observed in other Portuguese water estuaries, such as those of the Douro, Sado, Ave, and Minho Rivers [46,47], and at the natural park in Doñana Ana, Spain, where the DDT/∑DDE, DDD ratio was >1 [48].

Compared to 2017 [40], the concentrations of alachlor and metribuzin are now 27- and 12-fold lower than those reported earlier, but that of dimethoate increased 2.5-fold. The presence of alachlor and metribuzin in this estuary has been linked to rice and maize crops and vineyards [41]. In the Mondego estuary, where rice crops are also abundant, the levels of the last three pesticides were 14-fold lower for alachlor (≈100 ng/L) and approximately 3-fold higher for both metribuzin (≈50 ng/L) and dimethoate (160 ng/L) [49].

Comparing the levels of the present 56 pesticides measured here with those published for the Tagus estuary—which is located approximately ≈50 km north of Sado estuary—it is observed that the global levels of fungicides, herbicides, and insecticides were, respectively, 6-, 130-, and 10-fold higher in the Sado estuary [25,26]. Concerning the surface water limits, the present levels were 25-, 115-, and 7-fold higher in the Sado than in the Tagus estuary [25,27].

Overall, this study shows that 22 insecticides had concentrations above the EU legislation for drinking water [50], and all were above the limits established for surface waters [27]. Excessive usage of pesticides also occurs in other aquatic systems across the world, including many European habitats [25,49,51,52,53,54,55,56]. Similar observations were described in the United States [57,58] and worldwide [59].

In this study, site **A** had a higher concentration of all categories of pesticides than the other sampled locations. In that site, the fungicides, herbicides, and insecticides were 4-, 2-, and 3-fold higher than in the other areas. The difference between this site and the others may be seen in the network images, which show that region **A** has no links to the others. So, it is proposed that site A is a “hot spot” for the pollution occurring in the estuary before its drainage into the Atlantic Ocean (Figure 1).

No distinct seasonal fluctuation patterns were measured for pesticides, in line with the high compound concentration variability. However, when analyzing the compounds with the highest concentrations, fungicides and herbicides tend to be higher in spring, when they are usually applied in this region [25]. In contrast, insecticides dominate in summer, when they are commonly used in rice fields [60].

The PCA reveals that pesticides show higher variability in winter, likely due to rain floods that leach this basin, producing a wide distribution of all analyzed compounds. Considering each category of pesticides per se, the PCA shows that each one has predominant seasons when their variability in the basin is higher, likely coincident with their application period. Indeed, fungicides and herbicides dominate in spring, whereas insecticides are widely applied from spring to summer before being lixiviated in winter.

Concerning the possible impacts of the studied pesticides in a realistic worst-case scenario, the maximal concentration measured in the Sado River estuary was used to infer their toxic implications for aquatic life. From these data, it was observed that some pesticides attained environmental concentrations able to cause mortality to 50% (LC50) of the exposed population of invertebrates (crustaceans: The water flea *Daphnia magna* and the mysid shrimp *Americamysis bahia*) and fish (*Oncorhynchus mykiss*) [61]. Amongst the most prominent compounds are several insecticides such as azinphos-methyl, chlorpyriphos, cyfluthrin, cypermethrin-alpha, deltamethrin, and aldrin. Similar results were found in the regionally close Tagus estuary [25].

However, since these compounds are not isolated but in environmental mixtures, the final toxicological action can be much more harmful to the biota. Thus, this study applied a two-tiered approach to evaluate the impact of the present pesticide mixture considering three trophic levels (algae, invertebrates, and fish). The first tier demonstrated a potential risk, primarily for invertebrates that exhibit the highest sum of toxic units STU (388.8), a value dominated by the insecticides cyfluthrin and chlorpyrifos. The second tier represents the maximal value by which CA may predict toxicity higher than IA (by the IA/CA ratio) [32]. In this case, it is proposed that two compounds—likely cyfluthrin and chlorpyriphos—are responsible for local toxicity. Similar predictions were already calculated for Tagus and Mondego estuaries, despite the fish being the most affected trophic level in those habitats [25,49].

Because the theoretical approach pointed to invertebrates as the most sensitive trophic group to the current environmental pesticide mixture, in vivo assays used the invertebrate *D. magna* as a model to test that hypothesis. After 24 h and 48 h of exposure, the control groups had low average mortalities in both pesticide mixtures, one considering an average of the maximal concentrations of all pesticides and the other containing the maximal concentrations of cyfluthrin and chlorpyriphos. There were no significant differences between the two exposed groups. The in vivo observations support the theoretical approach by confirming toxicological impacts in both tested conditions.

Several physicochemical parameters can be linked to pollution, and this aspect was also considered here. The DO, pH, and nitrate values were within acceptable ranges, as defined by Portuguese and EU legislation [62], i.e., DO ≈ 7.2 mg/L, pH ranged from 7.7 to 8.4, and nitrates < 1.3 mg/L [62]. However, the phosphates—usually originating from WWTPs and excess organophosphorus pesticides—exceeded the recommended value of 0.1 mg/L, defined as a limit to prevent eutrophication [34]. Thus, despite being the target of several depollution measures, the data points out that the estuary is still impacted and eutrophicated by human activities.

This study shows that despite the national and EU regulations on the use of pesticides and tolerable environmental levels, chemical monitoring confirms the presence of legal and forbidden pesticides in the Sado estuary water in wide diversity and concentrations, ranging from low to very high and above the legally required maximums. Some physicochemical parameters also support excessive anthropogenic impacts. The theoretical risk assessment suggests toxicological implications for the biota, particularly for invertebrates. In vivo assays with Daphnia exposed to realistic mixtures unequivocally confirms the risks. Given that the Sado estuary is a critical habitat for many species, in addition to hosting human recreational activities and aquaculture, our research warns that the area requires regular chemical monitoring, the identification and elimination of pesticide inputs, and eventually the consideration of decontamination actions to meet EU Directives and promote good ecological status.

## Figures and Tables

**Table 1:** Average concentrations of fungicides, herbicides, and insecticides (ng/L) in surface waters collected in the Sado River region, southwest Atlantic Iberian seacoast (*n* = 54, mean ± standard deviation). Data are organized in alphabetical order and by season. For each pesticide, we also refer to its frequency of detection (%), the global frequency of detection by category, and the global amounts (∑average ± SD) by season.

Fungicides	MDL (ng/L)	Frequency	Environmental Levels (ng/L) ± Mean (SD)			
		(%)	Spring	Summer	Autumn	Winter
Azoxystrobin	2.8	100	342.4 ± 462.2	265.1 ± 386.6	315.3 ± 536.6	649.9 ± 1466
Difenoconazole	2.0	100	6170 ± 14,409	262.7 ± 485.5	268.4 ± 725.2	620.3 ± 1202
HCB	2.1	59	3.6 ± 4.8	2.6 ± 2.1	2.3 ± 2.1	3.9 ± 6.6
PCB	2.7	89	16.7 ± 17.6	17.8 ± 26.6	11.8 ± 19.8	6.9 ± 5.4
Procymidone	2.0	100	159.4 ± 360.1	355.5 ± 928.6	409.6 ± 967.8	915.2 ± 2171
Tebuconazole	1.8	100	238.4 ± 388.2	675.6 ± 1645	509.2 ± 1209	388.8 ± 829.4
**% Frequency > MDL**		**91**				
**Fungicides (** **Σ** **average)**			**6930 ± 5785**	**1579 ± 623**	**1517 ± 493**	**2585 ± 851**
**Herbicides**	**MDL (ng/L)**	**Frequency (%)**	**Environmental Levels (ng/L) ± Mean (SD)**			
			**Spring**	**Summer**	**Autumn**	**Winter**
Alachlor	1.5	87	8.0 ± 11.5	6.6 ± 11.9	7.6 ± 11.9	5.7 ± 8.5
Atrazine	2.1	68	11.4 ± 12.3	10.8 ± 9.0	7.4 ± 9.0	7.0 ± 8.5
Atrazine-desethyl	1.6	100	51.9 ± 41.1	57.3 ± 48.1	56.1 ± 48.1	37.2 ± 19.7
Cyanazine	1.9	100	95.0 ± 89.5	53.6 ± 74.8	61.5 ± 74.8	69.8 ± 92.3
Cyhalofop-butyl	2.9	98	471.3 ± 737.1	535.0 ± 790.9	406.4 ± 790.9	547.4 ± 1131
Metolachlor	3.1	23	3.1 ± 1.6	2.7 ± 1.9	2.9 ± 1.9	4.9 ± 7.7
Metribuzin	1.8	100	253.3 ± 179.8	58.6 ± 55.3	60.0 ± 55.3	157.5 ±283.0
Pendimethalin	1.4	100	5.8 ± 3.2	6.0 ± 7.8	7.2 ± 7.8	5.3 ± 4.4
Propazine	1.7	100	161.1 ± 218.2	45.4 ± 44.9	45.0 ± 44.9	54.7 ± 79.4
Propyzamide	1.8	100	57.5 ± 66.5	25.2 ± 35.3	30.9 ± 35.3	34.0 ± 39.6
Simazine	2.8	100	43.1 ± 52.1	37.9 ± 26.9	27.0 ± 26.9	43.5 ± 78.9
Simetryn	1.8	100	21.2 ± 22.6	20.1 ± 25.6	20.5 ± 25.6	23.2 ± 32.2
Terbuthylazine	1.5	100	202.0 ± 264.1	50.3 ± 60.0	56.8 ± 60.0	54.6 ± 68.0
Terbutryn	2.0	94	10.9 ± 14.2	8.8 ± 12.9	9.8 ± 12.9	10.8 ± 19.7
Trifluralin	2.2	42	3.1 ± 2.0	2.7 ± 2.0	3.0 ± 2.0	4.0 ± 5.7
**% Frequency > MDL**		**87**				
**Herbicides (** **Σ** **average)**			**1399 ± 192**	**921 ± 198**	**802 ± 198**	**1060 ± 287**
**Insecticides**	**MDL (ng/L)**	**Frequency (%)**	**Environmental Levels (ng/L) ± Mean (SD)**			
			**Spring**	**Summer**	**Autumn**	**Winter**
Aldrin	2.5	58	138.2 ± 283.9	91.1 ± 200.4	59.5 ± 102.4	280.1 ± 821.3
Azinphos-methyl	2.8	100	3082 ± 7649	2778 ± 7515	1673 ± 3839	172.2 ± 149.9
Chlordane γ	1.8	47	76.9 ± 189.9	41.0 ± 98.0	18.8 ± 31.7	9.6 ± 16.9
Chlorpyriphos	2.3	100	115.1 ± 286.1	92.3 ± 264.2	40.5 ± 82.6	375.8 ± 1100.6
Chlorfenvinphos	1.8	75	141.5 ± 338.9	65.2 ± 149.1	64.4 ± 132.5	349.5 ± 994.4
Cyfluthrine	1.9	100	1643 ± 4381	865.6 ± 2255	402.5 ± 880.0	3574 ± 10,439
Cyhalothrin-λ	2.1	100	558.2 ± 1512	348.7 ± 1193	124.3 ± 292.1	489.4 ± 1420
Cypermethrin-α	1.9	100	687.0 ± 1833	319.2 ± 884.1	218.7 ± 495.4	689.9 ± 1980
DDD	1.9	92	152.5 ± 401.4	72.8 ± 200.8	33.0 ± 69.9	710.5 ± 2100
DDE	1.6	53	37.1 ± 94.0	24.6 ± 58.1	19.3 ± 44.1	322.3 ± 955.0
DDT	2.4	72	518.3 ± 1218	79.9 ± 216.1	71.1 ± 185.9	470.0 ± 1357
Deltametrin	2.2	100	659.8 ± 1608	508.2 ± 1375	266.1 ± 543.0	1062 ± 3026
Diazinon	1.7	100	327.5 ± 478.7	222.1 ± 265.8	197.4 ± 281.2	447.9 ± 814.6
Dichlorvos	2.4	100	67.1 ± 60.7	60.3 ± 101.4	62.0 ± 74.1	33.0 ± 23.4
Dieldrin	2.0	100	153.7 ± 395.3	132.7 ± 286.3	163.4 ± 293.7	869.7 ± 2155.7
Dimethoate	2.6	100	349.0 ± 531.7	407.2 ± 707.7	441.9 ± 403.7	581.4 ± 971.6
Endosulfan-α	1.6	100	394.5 ± 976.3	214.8 ± 526.6	193.3 ± 440.2	693.4 ± 1990
Endosulfan-β	2.2	70	175.7 ± 361.1	82.8 ± 161.6	55.7 ± 100.8	377.0 ± 1035
Endosulfan-sulphate	2.1	100	1378 ± 2114	410.9 ± 1079	226.0 ± 532.0	272.4 ± 437.9
Endrin	2.0	100	368.8 ± 923.7	353.3 ± 1119	134.0 ± 251.6	1279 ± 3689
Fenamiphos	2.5	100	1466 ± 3413	2032 ± 6380	855.3 ± 1821	4986 ± 14,321
Fenitrothion	1.8	98	12.6 ± 5.6	17.4 ± 19.7	15.3 ± 21.7	15.2 ± 17.2
Fonofos	1.4	100	61.3 ± 36.4	104.7 ± 131.1	94.7 ± 85.6	92.8 ± 105.1
Heptachlor	2.3	51	5.0 ± 4.6	4.8 ± 4.5	5.2 ± 6.9	36.6 ± 89.9
Heptachlor-epoxide	1.6	60	193.2 ± 347.8	46.5 ± 109.5	19.0 ± 31.3	76.7 ± 139.6
HCCP	1.1	0	0.8 ± 0.0	0.8 ± 0.0	0.8 ± 0.0	0.8 ± 0.0
Lindane	2.3	85	47.5 ± 68.8	8.8 ± 7.7	7.5 ± 8.2	12.8 ± 16.5
Malathion	3.1	100	27.5 ± 21.5	18.0 ± 20.3	22.1 ± 27.8	27.3 ± 36.8
Methoxychlor	1.3	100	401.6 ± 712.9	257.6 ± 651.8	67.6 ± 165.8	265.3 ± 608.2
Mirex	1.8	96	733.8 ± 2034	228.2 ± 785.7	125.5 ± 314.5	554.4 ± 1621
Parathion-ethyl	2.6	64	9.2 ± 7.8	8.1 ± 14.0	8.7 ± 12.8	8.2 ± 13.8
Parathion-methyl	2.0	74	25.8 ± 21.8	24.1 ± 25.6	22.8 ± 18.8	16.9 ± 13.0
Phosmet	2.4	87	55.1 ± 28.7	84.4 ± 93.0	54.5 ± 55.9	54.2 ± 144.5
Pirimicarb	1.5	100	104.0 ± 89.9	77.3 ± 70.9	115.7 ± 156.1	147.1 ± 234.5
Tetrachlorvinphos	1.2	100	563.1 ± 1462	417.1 ± 1123	179.2 ± 389.4	621.2 ± 1739
**% Frequency > MDL**		**85**				
**Insecticides (** **Σ** **average)**			**14,731 ± 1541**	**10,499 ± 1625**	**6059 ± 696**	**19,974 ± 2897**

**Table 2:** Assessment of the average annual values measured for certain pesticides at Sado estuary, in which average levels surpass the yearly average values (ng/L) established by the European legislation (98/83/EC and 2013/39/EU).

EU Legislation	Pesticides		Sado Estuary Annual Average Values (ng/L)	Directive Annual Values (ng/L)	License	log K_ow_	log K_oc_	GUS Index
**Drinking waters** **Directive 98/83/EC**	**Fungicides**							
	Azoxystrobin		393	100	A	2.5	2.8	2.6
	Difenoconazole		1830	100	A	4.4	3.6	0.9
	Tebuconazole		460	100	A	3.7	3.7	2.0
	**Herbicides**							
	Cyhalofop-butyl		490	100	A	6.0	3.7	−0.2
	Metribuzin		132	100	A	1.7	1.8	2.6
	**Insecticides**							
	Aldrin		142	100	B	6.5	4.2	−0.4
	Azinphos-methyl		1926	100	NA	3.0	3.0	1.0
	Chlorpyriphos		156	100	A	4.7	3.9	0.2
	Chlorfenvinphos		155	100	NA	3.8	2.8	1.9
	Cyfluthrine		1621	100	A	5.6	4.8	−1.7
	Cyhalothrin-λ		380	100	A	6.8	5.2	−2.1
	Cypermethrin-α		479	100	A	6.9	4.4	−2.1
	DDD		242	100	B	6.9	4.7	−0.9
	DDE		101	100	B	6.9	4.9	−2.0
	DDT		285	100	B	6.9	5.9	−4.5
	Deltametrin		624	100	A	4.6	7.0	−3.4
	Diazinon		299	100	NA	3.7	2.8	1.1
	Dieldrin		330	100	B	3.7	4.4	−0.3
	Dimethoate		445	100	A	0.7	1.0	1.1
	Endosulfan		547	100	NA	4.8	4.2	−0.1
	Endosulfan-sulphate		572	100	NA	3.7	3.7	0.5
	Endrin		534	100	NA	3.2	4.0	0.0
	Fenamiphos		2335	100	A	3.3	2.0	−0.1
	Methoxychlor		248	100	NA	3.8	4.9	−1.9
	Mirex		410	100	B	5.3	3.8	0.6
	Pirimicarb		111	100	A	1.7	2.6	2.7
	Tetrachlorvinphos		445	100	NA	3.5	3.0	0.3
	Σ_Aldrin,Dieldrin,Heptachlor,Heptachlor epoxide_		1006	100	B; B; B; NA	5.2	4.3	−0.7
**Concentration (average) of the pesticides above 98/83/EC**			**596**					
**EU Legislation**	**Pesticides**		**Sado estuary Annual average values (ng/L)**	**Directive Annual values (ng/L)**	**License**	**log K** **_ow_**	**log K** **_oc_**	**GUS index**
**Surface waters** **Directive 2013/39/EU**	**Fungicides**							
	HCP		3	0.02	-	4.0	3.6	0.4
	PCB		13	7	NA	4.8–5.2	4.5	−1.2
	**Herbicides**							
	Alachor		7	300	NA	3.7	2.5	0.8
	Atrazine		9	600	NA	2.7	2.0	3.3
	Simazine		38	1000	NA	2.3	2.1	2.0
	Terbutryn		10	65 ^a^–0.65 ^b^	NA	3.7	3.4	2.4
	Trifluralin		3	30	NA	5.3	4.2	0.1
	**Insecticides**							
	Chlorfenvinphos		155	100	A	4.7	3.9	0.2
	Cypermethrin		242	0.08 ^a^–0.008 ^b^	A	6.9	4.4	−2.1
	4.4’-DDD		547	25	B	6.9	4.7	−0.9
	Dichlorvos		479	0.6 ^a^–0.06 ^b^	NA	1.9	1.7	0.7
	Endosulfan		56	5 ^a^–0.5 ^b^	NA	4.7–4.8	4.1–4.3	−0.1
	Heptachlor		13	2 × 10^−4 a^–1 ×10^−4 b^	B	5.4	4.4	−0.9
	Heptachlor epoxide		84	2 × 10^−4 a^–1 × 10^−4b^	NA	4.4–5.5	4.3	−1.1
	ΣAldrin. Dieldrin. Endrin. Isodrin *		1006	5 ^a–^10 ^b^	B; B; NA; -	4.5	4.2	−0.2
**The average concentration above 2013/39/EU**			**178**					
	^a^	Inland surface waters	Licence according to the EU Pesticides Database: NA—authorised; A—Authorised; B—Banned					
	^b^	Other surface waters	GUS index (groundwater ubiquity score; GUS = log10 (half life-days) × [4 − log10 (Koc)])					
	*		Isodrin was not evaluated in this study					

**Table 3:** Ecological hazard assessment of pesticides in the Sado River estuary. The concentrations referred to herein are the maximal environmental concentrations (MECs) measured in surface water collected from this habitat.

	Pesticides	MEC (mg/L)	Algae 72 h EC_50_ Growth (mg/L)	Invertebrates 48 h EC_50_ (mg/L)	Fish 96 h LC_50_ (mg/L)	PNEC (mg/L)	Individual RQ (MEC/PNEC)	RQ TU Algae (MEC/EC_50_)		RQ TU Invert. (MEC/EC_50_)	RQ TU Fish (MEC/EC_50_)
**FUNGICIDES**	Azoxystrobin	4.5 × 10^−3^	3.6 × 10^−1^	2.3 × 10^−1^	4.7 × 10^−1^	2.3 × 10^−3^	2.0	0.0		0.0	0.0
	Difenoconazol	4.2 × 10^−2^	3.2 × 10^−2^	7.7 × 10^−1^	1.1 × 10^0^	3.2 × 10^−4^	130.4	1.3		0.1	0.0
	HCB	2.2 × 10^−5^	1.0 × 10^−2^	5.0 × 10^−1^	3.0 × 10^−2^	1.0 × 10^−4^	0.2	0.0		0.0	0.0
	PeCB	1.0 × 10^−4^	1.3 × 10	-	2.5 × 10^−1^	2.5 × 10^−3^	0.0	0.0		-	0.0
	Procymidone	6.6 × 10^−3^	2.6 × 10	1.8 × 10^0^	7.2 × 10^0^	1.8 × 10^−2^	0.4	0.0		0.0	0.0
	Tebuconazole	5.4 × 10^−3^	2.0 +	2.8 × 10^0^	4.4 × 10^0^	2.0 × 10^−2^	0.3	0.0		0.0	0.0
**HERBICIDES**	Alachlor	5.2 × 10^−5^	9.7 × 10^−1^	1.0 × 10	1.8 × 10^0^	9.7 × 10^−3^	0.0	0.0		0.0	0.0
	Atrazine	5.8 × 10^−5^	5.9 × 10^−2^	8.5 × 10	4.0 × 10^0^	5.9 × 10^−4^	0.1	0.0		0.0	0.0
	Atrazine-desethyl	1.9 × 10^−4^	1.0 × 10^−1^	-	-	1.0 × 10^−3^	0.2	0.0		-	-
	Cyanazine	3.2 × 10^−4^	2.0 × 10^−1^	4.9 × 10	1.0 × 10^1^	2.0 × 10^−3^	0.2	0.0		0.0	0.0
	Cyhalofop-butyl	4.9 × 10^−3^	9.6 × 10^−1^	2.7 × 10^0^	7.9 × 10^−1^	7.9 × 10^−3^	0.6	0.0		0.0	0.0
	Metolachlor	2.5 × 10^−5^	5.7 × 10^1^	2.4 × 10	3.9 × 10^0^	3.9 × 10^−2^	0.0	0.0		0.0	0.0
	Metribuzin	8.7 × 10^−4^	2.0 × 10^−2^	4.9 × 10	7.5 × 10^1^	2.0 × 10^−4^	4.3	0.0		0.0	0.0
	Pendimethalin	3.2 × 10^−5^	6.0 × 10^−3^	2.8 × 10^−1^	1.4 × 10^−1^	6.0 × 10^−5^	0.5	0.0		0.0	0.0
	Propazine	6.9 × 10^−4^	1.8 × 10^−1^	1.8 × 10	1.8 × 10^1^	1.8 × 10^−3^	0.4	0.0		0.0	0.0
	Propyzamide	1.9 × 10^−4^	2.8 × 10	5.6 × 10^0^	4.7 × 10^0^	2.8 × 10^−2^	0.0	0.0		0.0	0.0
	Simazine	2.5 × 10^−4^	4.0 × 10^−2^	1.1 × 10^0^	9.0 × 10^1^	4.0 × 10^−4^	0.6	0.0		0.0	0.0
	Simetryn	1.1 × 10^−4^	9.8 × 10^−3^	-	7.0 × 10^0^	9.8 × 10^−5^	1.1	0.0		-	0.0
	Terbuthylazine	8.4 × 10^−4^	1.2 × 10^−2^	2.1 × 10	2.2 × 10^0^	1.2 × 10^−4^	7.0	0.1		0.0	0.0
	Terbutryn	6.3 × 10^−5^	2.4 × 10^−3^	2.7 × 10^0^	1.1 × 10^0^	2.4 × 10^−5^	2.6	0.0		0.0	0.0
	Trifluralin	1.9 × 10^−5^	1.2 × 10^−2^	2.5 × 10^−1^	8.8 × 10^−2^	1.2 × 10^−4^	0.2	0.0		0.0	0.0
**INSECTICIDES**	Aldrin	2.5 × 10 ^−3^	-	2.8 × 10^−2^	4.6 × 10^−3^	4.6 × 10^−5^	53.7	-		0.1	0.5
	Azinphos-methyl	2.4 × 10^−2^	7.2 × 10^0^	1.1 × 10^−3^	2.0 × 10^−2^	1.1 × 10^−5^	2201.7	0.0		22.0	1.2
	Chlordane-γ	5.4 × 10^−4^	-	5.9 × 10^−1^	9.0 × 10^−2^	9.0 × 10^−4^	0.6	-		0.0	0.0
	Chlorpyriphos	3.3 × 10-^3^	4.8 × 10^−1^	4.0 × 10^−5^	1.3 × 10^−3^	4.0 × 10^−7^	8276.7	0.0		**82.8**	2.5
	Chlorfenvinphos	3.0 × 10^−3^	1.4 × 10^0^	2.5 × 10^−4^	1.1 × 10^0^	2.5 × 10^−6^	1200.3	0.0		12.0	0.0
	Cyfluthrin	3.1 × 10^−2^	1.0 × 10	1.6 × 10^−4^	4.7 × 10^−4^	1.6 × 10^−6^	19,631.9	0.0		**196.3**	66.8
	Cyhalothrin- γ	4.9 × 10^−3^	-	3.8 × 10^−1^	4.6 × 10^−4^	4.6 × 10^−6^	1064.6	-		0.0	10.6
	Cypermethrin-α	6.0 × 10^−3^	1.0 × 10^−1^	3.0 × 10^−4^	2.8 × 10^−3^	3.0 × 10^−6^	1990.2	0.1		19.9	2.1
	Σ_DDD,DDE,DDT_	1.3 × 10^−2^	-	5.0 × 10^−3^	7.0 × 10^0^	5.0 × 10^−5^	265.3	-		2.7	0.0
	Deltametrin	9.1 × 10^−3^	9.1 × 10^0^	5.6 × 10^−4^	2.6 × 10^−4^	2.6 × 10^−6^	3511.8	0.0		16.3	35.1
	Diazinon	2.6 × 10^−3^	6.4 × 10^0^	1.0 × 10^−3^	3.1 × 10^0^	1.0 × 10^−5^	260.2	0.0		2.6	0.0
	Dichlorvos	4.5 × 10^−4^	5.3 × 10	1.9 × 10^−4^	5.5 × 10^−1^	1.9 × 10^−6^	236.3	0.0		2.4	0.0
	Dieldrin	6.6 × 10^−3^	1.0 × 10^−1^	2.5 × 10^−1^	1.2 × 10^−3^	1.2 × 10^−5^	549.7	0.1		0.0	5.5
	Dimethoate	3.0 × 10^−3^	9.0 × 10	2.0 × 10^0^	3.0 × 10	2.0 × 10^−2^	0.1	0.0		0.0	0.0
	Endosulfan (α + β)	9.1 × 10^−3^	2.2 × 10^0^	4.4 × 10^−1^	2.0 × 10^−3^	2.0 × 10^−5^	456.5	0.0		0.0	4.6
	Endosulfan sulfate	5.9 × 10^−3^	-	-	-	-	-	-		-	-
	Endrin	1.1 × 10^−2^	-	4.20 × 10^−3^	7.3 × 10^−4^	7.3 × 10^−6^	1250.8	-		2.2	12.5
	Fenamiphos	4.3 × 10^−2^	3.8 × 10^0^	1.90 × 10^−3^	9.3 × 10^−3^	1.9 × 10^−5^	2272.0	0.0		22.7	4.6
	Fenitrothion	9.1 × 10^−5^	1.3 × 10^0^	8.60 × 10^−3^	1.3 × 10^0^	8.6 × 10^−5^	1.1	0.0		0.0	0.0
	Fonofos	5.3 × 10^−4^	1.5 × 10^0^	2.30 × 10^−3^	2.8 × 10^−2^	2.3 × 10^−5^	23.0	0.0		0.2	0.0
	Heptachlor	2.7 × 10^−4^	2.7 × 10^−2^	4.20 × 10^−2^	7.0 × 10^−3^	7.0 × 10^−5^	3.9	0.0		0.0	0.0
	Heptachlor epoxide	9.2 × 10^−4^	2.0 × 10^2^	2.40 × 10^−1^	2.0 × 10^−2^	2.0 × 10^−4^	4.6	0.0		0.0	0.0
	HCCP	8.0 × 10^−7^	-	5.20 × 10^−2^	2.4 × 10^0^	5.2 × 10^−4^	0.0	-		0.0	0.0
	Lindane	2.1 × 10^−4^	2.5 × 10^0^	1.60 × 10^0^	2.9 × 10^−3^	2.9 × 10^−5^	7.4	0.0		0.0	0.1
	Malathion	1.2 × 10^−4^	1.3 × 10	7.0 × 10^−4^	1.8 × 10^−2^	7.0 × 10^−6^	17.4	0.0		0.2	0.0
	Methoxychlor	2.6 × 10^−3^	6.0 × 10^−1^	7.8 × 10^−4^	5.2 × 10^−2^	7.8 × 10^−6^	329.8	0.0		3.3	0.0
	Mirex	5.8 × 10^−3^	1.0 × 10^−1^	1.0 × 10^−1^	1.0 × 10^2^	1.0 × 10^−3^	5.8	0.1		0.1	0.0
	∑_Parathion-methyl. ethyl_	1.5 × 10^−4^	3.0 × 10^0^	7.3 × 10^−3^	2.7 × 10^0^	7.3 × 10^−5^	2.0	0.0		0.0	0.0
	Phosmet	4.4 × 10^−4^	7.0 × 10^−2^	2.0 × 10^−3^	2.3 × 10^−1^	2.0 × 10^−5^	21.9	0.0		0.2	0.0
	Pirimicarb	7.7 × 10^−4^	1.4 × 10^2^	1.7 × 10^−2^	1.0 × 10^2^	1.7 × 10^−4^	4.5	0.0		0.0	0.0
	Tetrachlorvinphos	5.3 × 10^−3^	-	2.0 × 10^−3^	4.3 × 10^−1^	2.0 × 10^−5^	262.9	-		2.6	0.0
					**∑RQ_MEC/PNEC_**		**44,058**				
				**First-tier (CA-based)**				1.7		**388.8**	146.6
						**RQ_STU_**				38,875	
				**Second-tier (IA-based)**			**maxRQSTU/maxTU**			**2.0**	

Note: $PNEC=\frac{L\left(E\right)C50}{AF=100}$ and $RQ=\frac{MEC}{PNEC}$.

**Table 4:** Physicochemical parameters of water samples from Sado estuary (mean ± SD).

Season	Sites	T (°C)	pH	DO (mg/L)	Salinity (PSU)	Nitrites (mg/L)	Nitrates (mg/L)	Ammonium (mg/L)	Phosphates (mg/L)
Spring (*n* = 5)	**A**–**E**	16.9 ± 1.1	8.0 ± 0.3	7.8 ± 2.0	31.1 ± 3.7	0.02 ± 0.0	0.2 ± 0.1	0.6 ± 0.5	0.6 ± 1.9
Summer (*n* = 10)		20.4 ± 1.9	8.1 ± 0.2	6.6 ± 2.4	34.8 ± 1.3	0.04 ± 0.1	0.2 ± 0.2	0.6 ± 0.6	1.2 ± 1.4
Autumn (*n* = 10)		14.1 ± 2.3	8.4 ± 0.2	8.2 ± 1.0	31.0 ± 5.4	0.02 ± 0.0	0.4 ± 0.2	1.1 ± 1.5	0.7 ± 1.0
Winter (*n* = 5)		12.7 ± 2.6	8.2 ± 0.1	9.3 ± 0.5	28.8 ± 5.6	0.03 ± 0.0	0.4 ± 0.2	0.5 ± 0.4	0.01 ± 0.0
Spring (*n* = 4)	**F**–**I**	17.6 ± 0.4	7.7 ± 0.3	6.0 ± 1.8	16.9 ± 13.8	0.03 ± 0.0	1.0 ± 0.5	0.8 ± 0.6	0.5 ± 0.3
Summer (*n* = 8)		22.3 ± 2.1	7.9 ± 0.3	5.4 ± 1.9	12.8 ± 14.7	0.07 ± 0.1	1.3 ± 2.6	0.7 ± 0.9	3.0 ± 1.6
Autumn (*n* = 8)		12.6 ± 3.6	8.1 ± 0.3	7.6 ± 1.4	10.0 ± 13.1	0.06 ± 0.0	1.0 ± 0.7	0.4 ± 0.3	0.8 ± 0.5
Winter (*n* = 4)		11.2 ± 0.5	7.9 ± 0.4	6.5 ± 4.8	12.8 ± 13.5	0.07 ± 0.0	0.2 ± 0.4	0.4 ± 0.2	0.3 ± 0.6

## Data Availability

The data presented in this study are available from the corresponding author on reasonable request.

## Supplementary Materials

The following supporting information can be downloaded at: https://www.mdpi.com/article/10.3390/toxics11050457/s1. Table S1: Quantification and diagnostic ions used in GC-MS/MS analyses. The relative abundance of ions (m/z) for each target pesticide is between brackets. Table S2: Data referring to the statistics on Figure 2. Table S3: Data referring to the PCAs of fungicides, herbicides and insecticides.
