# Supplementary material for: Chemical Survey and Risk Assessment of 56 Pesticides in the Sado River Estuary (Iberian Peninsula, Portugal)
Source: Toxics. 2023 May 14;11(5):457. doi: 10.3390/toxics11050457 (PMC10221532; doi:10.3390/toxics11050457)
Supplement: Supplementary file 1 [file toxics-11-00457-s001.zip › Table S1.pdf]

Table S1 – Quantification and diagnostic ions used in GC-MS/MS analyses. The relative abundance of ions (*m/z*) for each target pesticide is between brackets.

Pesticides	Molecular mass g/mol	RT (min)	Target ion (t)	Q1 (%Q1/t)	Q2 (%Q2/t)	Q3 (%Q3/t)	Q 4 (%Q4/t)	Percursor		Products				EV	Ranges
**PeCB**	250.3	9.55	**250**	248 (65.1)	252 (66.1)			**250**	→	215	144			2.01	143-251
**Trifluralin**	335.3	10.90	**264**	306 (44.9)	206 (27.2)			**264**	→	206	160	188	171	1.05	159-265
**Atrazine-desethyl**	187.6	10.93	**172**	68 (32.2)	174 (29.2)			**172**	→	130	145	152		1.15	104-173
**Propazine**	229.7	11.66	**214**	172 (70.6)	187 (38.0)			**214**	→	200	172	138		1.20	137-215
**HCB** [b]	284.8	11.68	**284**	282 (46.4)	249 (41.2)			**284**	→	214	249			1.50	211-285
**Dimethoate**	229.3	11.77	**87**	93 (52.5)	125 (44.3)			**87**	→	86	59			1.10	53-88
**Simazine** [a]	201.6	11.88	**201**	186 (67.1)	173 (51.9)	44 (37.6)		**201**	→	186	174	138		1.20	135-201
**<u>ATZ-d$_5$</u>**	220.7	11.95	**205**	220 (43.0)	178 (41.7)			***205***	→	127	137	105		1.25	104-206
**Atrazine** [b]	215.7	12.00	**200**	215 (56.8)	173 (36.9)			**200**	→	122	132	164	158	1.25	121-201
**Lindane** [a]	290.8	12.33	**181**	183 (76.6)	219 (69.3)			**181**	→	145	146			1.20	108-184
**Terbuthylazine**	229.7	12.41	**214**	173 (71.8)	138 (27.3)	229 (24.3)		**214**	→	173	132			1.40	131-215
**Propyzamide**	256.1	12.45	**173**	175 (41.2)	254 (35.6)			**173**	→	138	145			1.10	130-174
**Diazinon**	304.4	12.46	**137**	179 (44.7)	304 (10.2)			**179**	→	121	163	137	122	1.35	110-180
**Fonofos**	246.3	12.51	**137**	109 (70.7)	246 (40.0)			**137**	→	109	81			0.85	80-138
**Pirimicarb**	238.4	13.10	**166**	238 (29.0)	72 (18.8)			**166**	→	96	137	121		1.35	95-167
**Parathion-methyl**	263.2	13.98	**263**	109 (67.9)	79 (44.7)	246 (43.5)		**263**	→	246	153			1.31	150-264
**Alachlor** [a]	269.8	14.01	**188**	160 (86.8)	146 (58.8)			**160**	→	132	130			1.10	116-161
**Simetryn**	213.3	14.10	**213**	170 (22.9)	155 (13.4)			**213**	→	170	185			1.10	151-214
**Heptachlor** [b]	373.3	14.25	**272**	274 (73.9)	270 (63.3)	100 (43.9)		**272**	→	237	235			1.05	236-275
**Metribuzin**	214.3	14.43	**198**	199 (29.8)				**198**	→	150	110			1.10	109-199
**Terbutryn**	241.4	14.67	**185**	226 (68.0)	170 (45.2)			**185**	→	170	128			0.90	127-186
**Fenitrothion**	277.2	14.71	**260**	109 (83.4)	125 (77.4)	277 (41.8)		**260**	→	228	217	232		1.20	160-261
**Malathion**	330.4	14.95	**125**	127 (90.9)	99 (73.2)	173 (40.2)		**173**	→	99	117	145		0.80	92-173
**Metolachlor**	283.8	15.12	**162**	238 (36.7)	163 (15.1)			**162**	→	132	133			1.15	115-163
**Chlorpyriphos**	350.6	15.19	**314**	316 (72.3)	258 (67.3)	199 (46.8)	197 (41.4)	**314**	→	258	286			0.90	257-315
**Cyanazine**	240.7	15.26	**225**	212 (59.4)	198 (35.2)	68 (32.8)		**225**	→	189	172	198		1.28	171-226
**Aldrin** [a]	364.9	15.35	**263**	261 (92.7)	265 (65.6)	66 (57.2)		**263**	→	193	191	227		1.60	190-264
**Parathion-ethyl**	291.3	15.38	**291**	109 (79.0)	263 (61.0)	97 (57.2)	141 (47.0)	**109**	→	81	91			0.99	60-110
**Pendimethalin**	281.3	16.27	**252**	162 (61.3)	191 (28.9)			**252**	→	162	191			1.00	160-253
**Chlorfenvinphos Z**	359.6	16.53	**267**	269 (52.9)	323 (51.5)			**267**	→	159	203			1.50	158-268
**Heptachlor epoxid**	389.3	16.53	**353**	355 (66.0)	351 (44.4)	81 (25.7)		**353**	→	263	282			1.10	262-354
**Procymidone**	284.1	16.86	**96**	283 (85.3)	285 (29.0)			**96**	→	67	68			1.00	64-97
**Chlordane (gamma**	338.9	17.28	**375**	373 (93.8)	377 (59.9)			**373**	→	266	264			1.20	263-374
**Tetrachlorvinphos**	366.0	17.40	**329**	331 (90.4)	109 (51.3)	333 (32.8)		**329**	→	314	278			1.30	219-330
**Endosulfan (beta)**	406.9	17.70	**241**	195 (72.7)	243 (71.2)	207 (54.0)		**241**	→	206	204	170		1.45	165-242
**Fenamiphos**	303.4	17.87	**303**	243 (62.4)	217 (54.9)	288 (42.9)	154 (40.6)	**303**	→	268	266			1.10	175-304
**4,4'-DDE**	318.0	18.40	**246**	248 (58.6)	318 (31.9)	316 (29.3)		**246**	→	176	175			1.70	174-247
**Dieldrin** [a]	380.9	18.60	**79**	263 (93.1)	237 (43.5)			**79**	→	51	50			1.10	49-80
**Endosulfan (alpha**	406.9	18.60	**241**	195 (78.2)	237 (70.8)	243 (65.5)		**241**	→	206	205			1.45	165-242
**Endrin** [b]	380.9	18.61	**243**	263 (99.0)	281 (68.4)	81 (47.4)		**243**	→	207	173			1.15	172-244
**4,4´-DDD**	320.0	19.84	**235**	237 (64.2)	165 (61.7)			**235**	→	165	199			1.15	162-236
**Endosulfan sulfate**	422.9	20.95	**272**	237 (68.0)	274 (60.5)	387 (47.9)		**272**	→	237	235			1.10	234-273
**<u>DDT-d$_8$</u>**	362.5	21.03	**220**	243 (62.6)	280 (57.8)			***243***	→	173	206			1.15	172-244
**4,4´-DDT**	354.5	21.11	**235**	237 (64.2)	212 (59.0)	165 (44.0)		**235**	→	165	199			1.15	117-236
**Methoxychlor** [b]	345.7	23.08	**227**	228 (16.5)	274 (15.4)			**227**	→	169	181			*1.30*	*140-228*
**Azinphos-methyl**	317.3	23.85	**77**	132 (88.0)	104 (43.4)	160 (32.9)		**77**	→	51	50			1.30	49-78
**Tebuconazole**	307.8	24.44	**250**	125 (80.6)	163 (44.4)			**125**	→	89	99			1.60	62-126
**Cyhalofop-butyl**	357.4	24.44	**256**	357 (72.6)	229 (41.1)	120 (31.0)		**256**	→	228	200			1.13	199-257
**Mirex**	545.5	24.71	**272**	274 (73.3)	237 (62.7)			**272**	→	237	235			1.13	234-273
**Cyhalothrin (lamb**	449.9	24.73	**181**	141 (45.8)	197 (42.1)			**181**	→	152	151			1.50	120-182
**Cyfluthrine (beta)**	434.3	27.71	**206**	199 (76.9)	91 (70.9)	226 (55.0)	227 (42.2)	**199**	→	193	191	163		1.80	190-200
**Cypermethrin (alp**	416.3	28.24	**181**	91 (76.3)	163 (75.0)	165 (47.3)		**181**	→	152	151			1.70	150-153/179-182
**Difenoconazol**	406.3	31.25	**265**	267 (89.9)	323 (68.5)	325 (62.2)		**323**	→	265	249			1.35	245-266/321-324
**Deltamethrin ***	505.2	32.00	**181**	207 (61.4)	253 (58.7)			**181**	→	152	151			1.70	150-153/179-182

<u>Internal standards</u> ; [a] Compounds present on the mix A (EPA 505/525); [b] Compounds present on the mix B (EPA 505/525); [*] Contain several diastereoisomers
