# Supplementary material for: Chemical Survey and Risk Assessment of 56 Pesticides in the Sado River Estuary (Iberian Peninsula, Portugal)
Source: Toxics. 2023 May 14;11(5):457. doi: 10.3390/toxics11050457 (PMC10221532; doi:10.3390/toxics11050457)
Supplement: Supplementary file 1 [file toxics-11-00457-s001.zip › Table S2.pdf]

Table S2 - Data referring to the statistics on **Figure 2**.

FUNGICIDES

	A	B	C	D	E	F	G	H	I
**A**									
**B**	1,1E-12								
**C**	1,8E-12								
**D**	1,1E-12								
**E**	8,1E-11			2,8E-02					
**F**	1,1E-12				1,1E-03				
**G**	1,1E-12	1,6E-08	1,4E-10	5,1E-08	2,1E-12	2,0E-06			
**H**	1,3E-09	6,2E-03		2,1E-03		6,1E-05	1,2E-12		
**I**	3,3E-11						2,1E-09		

HERBICIDES

	A	B	C	D	E	F	G	H	I
**A**									
**B**	1,9E-10								
**C**	1,3E-08								
**D**	8,0E-10								
**E**	2,9E-07								
**F**	7,0E-11								
**G**	7,2E-10								
**H**		2,4E-06	1,9E-04	1,1E-05	3,8E-03	8,2E-07	1,0E-05		
**I**	1,2E-06							5,1E-03	

INSECTICIDES

	A	B	C	D	E	F	G	H	I
**A**									
**B**	1,9E-10								
**C**	1,3E-08								
**D**	8,0E-10								
**E**	2,9E-07								
**F**	7,0E-11								
**G**	7,2E-10								
**H**		2,4E-06	1,9E-04	1,1E-05	3,8E-03	8,2E-07	1,0E-05		
**I**	1,2E-06							5,1E-03	
