# Supplementary material for: Chemical Survey and Risk Assessment of 56 Pesticides in the Sado River Estuary (Iberian Peninsula, Portugal)
Source: Toxics. 2023 May 14;11(5):457. doi: 10.3390/toxics11050457 (PMC10221532; doi:10.3390/toxics11050457)
Supplement: Supplementary file 1 [file toxics-11-00457-s001.zip › Table S3.pdf]

Table S3 – Data referring to the PCAs of fungicides, herbicides and insecticides.

**Fungicides**	PC 1	PC 2				
Azoxystrobin	0.41	-0.41				
Difenoconazole	0.21	0.82				
Hexachlorobenzene	0.46	0.14				
Pentachlorobenzene	0.39	0.26				
Procymidone	0.48	-0.25				
**Herbicides**	PC 1	PC 2	PC 3			
Alachlor	0.28	-0.12	0.20			
Atrazine	0.24	-0.28	0.20			
Atrazine-desethyl	0.23	-0.36	0.33			
Cyanazine	0.30	0.03	0.03			
Cyhalofop-butyl	0.27	-0.24	-0.09			
Metolachlor	0.24	0.10	-0.48			
Metribuzin	0.23	0.41	-0.21			
Pendimethalin	0.26	-0.32	0.09			
Propazine	0.24	0.43	0.28			
Propyzamide	0.28	0.16	0.14			
Simazine	0.27	-0.06	-0.19			
Simetryn	0.29	-0.14	-0.07			
Terbuthylazine	0.23	0.43	0.37			
Terbutryn	0.27	0.10	-0.10			
**Insecticides**	PC 1	PC 2	PC 3	PC 4	PC 5	PC 6
Aldrin	0.19	-0.16	-0.05	-0.01	-0.02	0.00
Azinphos-methyl	0.13	0.36	0.05	-0.10	-0.15	0.00
Chlordane-gamma	0.14	0.32	-0.17	-0.13	-0.11	0.00
Chlorpyriphos	0.19	-0.17	-0.03	0.00	-0.05	0.00
Chlorfenvinphos Z	0.20	-0.17	-0.03	0.00	0.04	0.00
Cyfluthrine	0.20	-0.14	-0.07	-0.01	-0.01	0.00
Cyhalothrin-lambda	0.19	0.08	-0.16	-0.09	-0.15	0.00
Cypermethrin-alpha	0.20	0.04	-0.13	-0.07	-0.03	0.00
DDD	0.18	-0.24	-0.02	0.03	0.03	0.00
DDE	0.17	-0.27	0.01	0.04	0.03	0.00
DDT	0.19	-0.04	-0.23	-0.03	0.08	0.00
Deltamethrin	0.21	-0.02	0.02	-0.02	-0.06	0.00
Diazinon	0.20	-0.02	0.00	-0.01	0.13	0.00
Dichlorvos	0.06	0.13	0.01	-0.21	0.82	0.00
Dieldrin	0.19	-0.22	0.03	0.02	0.03	0.00
Dimethoate	0.17	0.03	0.11	-0.08	-0.04	0.00
Endosulfan-alfa	0.21	-0.08	-0.02	-0.02	0.02	0.00
Endosulfan-beta	0.19	-0.19	-0.06	0.00	0.03	0.00
Endosulfan-sulphate	0.14	0.33	-0.16	0.12	-0.01	0.00
Endrin	0.19	-0.16	-0.03	0.00	-0.08	0.00
Fenamiphos	0.19	-0.11	0.02	-0.01	-0.14	0.00
Fenitrothion	0.14	0.12	0.37	0.02	-0.13	0.00
Fonofos	0.16	0.12	0.36	-0.01	-0.12	0.00
Heptachlor	0.03	0.02	-0.03	0.79	0.10	0.00
Heptachlor epoxide	0.11	0.21	-0.16	0.45	0.02	0.00
Hexachlorocyclopentadiene	0.00	0.00	0.00	0.00	0.00	1.00
Lindane	0.12	0.20	-0.39	-0.01	0.18	0.00

Malathion	0.17	0.07	0.23	0.13	0.21	0.00
Methoxychlor	0.18	0.15	-0.11	0.00	-0.18	0.00
Mirex	0.19	0.06	-0.25	-0.09	-0.02	0.00
Parathion-ethyl	0.17	0.16	0.27	0.13	0.04	0.00
Parathion-methyl	0.12	0.30	0.19	-0.13	0.05	0.00
Phosmet	0.17	0.01	0.33	0.03	0.04	0.00
Pirimicarb	0.17	-0.05	0.17	0.02	0.21	0.00
